# RNA sequencing-based cell proliferation analysis across 19 cancers identifies a subset of proliferation-informative cancers with a common survival signature

**DOI:** 10.18632/oncotarget.16961

**Published:** 2017-04-08

**Authors:** Ryne C. Ramaker, Brittany N. Lasseigne, Andrew A. Hardigan, Laura Palacio, David S. Gunther, Richard M. Myers, Sara J. Cooper

**Affiliations:** ^1^ HudsonAlpha Institute for Biotechnology, Huntsville, AL, USA; ^2^ Department of Genetics, University of Alabama at Birmingham, Birmingham, AL, USA

**Keywords:** cell proliferation, cancer, reelin, survival, RNA-seq

## Abstract

Despite advances in cancer diagnosis and treatment strategies, robust prognostic signatures remain elusive in most cancers. Cell proliferation has long been recognized as a prognostic marker in cancer, but the generation of comprehensive, publicly available datasets allows examination of the links between cell proliferation and cancer characteristics such as mutation rate, stage, and patient outcomes. Here we explore the role of cell proliferation across 19 cancers (*n* = 6,581 patients) by using tissue-based RNA sequencing data from The Cancer Genome Atlas Project and calculating a ‘proliferative index’ derived from gene expression associated with Proliferating Cell Nuclear Antigen (PCNA) levels. This proliferative index is significantly associated with patient survival (Cox, *p*-value < 0.05) in 7 of 19 cancers, which we have defined as “proliferation-informative cancers” (PICs). In PICs, the proliferative index is strongly correlated with tumor stage and nodal invasion. PICs demonstrate reduced baseline expression of proliferation machinery relative to non-PICs. Additionally, we find the proliferative index is significantly associated with gross somatic mutation burden (Spearman, *p* = 1.76 × 10−23) as well as with mutations in individual driver genes. This analysis provides a comprehensive characterization of tumor proliferation indices and their association with disease progression and prognosis in multiple cancer types and highlights specific cancers that may be particularly susceptible to improved targeting of this classic cancer hallmark.

## INTRODUCTION

A fundamental characteristic of cancer cells is their ability to maintain the capacity to proliferate, bypassing the homeostatic signaling network controlling cell division in normal tissue. The capacity to “sustain proliferative signaling”, “enable replicative immortality”, and “evade growth suppressors” represent three of the original six hallmarks of cancer, and histological techniques examining the number of mitotic cells present in tumor biopsies have been used clinically to assess tumor grade for several decades [1, 2]. Although proliferation is a clear hallmark of cancer, tumor evolutionary tradeoffs may exist in certain tumor types or stages that prioritize resources for other processes promoting survival like metastasis [3, 4], angiogenesis [5–7], immune system evasion [8, 9], drug efflux [10, 11], DNA repair [12, 13], drug resistance [14], or reactive oxygen species (ROS) regulation [15]. Characterizing these tradeoffs is critical to achieving a complete understanding of tumor progression and selecting appropriate therapies [16].

Early studies comparing tumor with adjacent normal tissue identified expression changes in genes controlling cell proliferation as some of the largest and most consistent cancer alterations and further associated proliferation signatures with poor patient prognosis and advanced tumor grade [17–22]. More recently, large-scale sequencing efforts have described driver mutations that hijack normal proliferation machinery. For example, approximately 40% of melanomas carry activating *BRAF* mutations which modulate proliferation by constitutively activating the downstream mitogen activated protein kinase (*MAPK*) pathway [23]. Multiple tumor types also harbor activating mutations in phosphoinositide 3-kinase (*PI3K*) that hyperactivate *AKT/mTOR* signaling and several other pathways important for regulating proliferation [24]. Accordingly, a majority of cytotoxic chemotherapies preferentially target the increased proliferation rate of cancer cells by damaging DNA in dividing cells or impairing vital replication machinery [25, 26].

Venet et al. derived a general index of proliferation, ‘metaPCNA’, by identifying the top 1% of genes most positively correlated with the proliferation marker *PCNA* (proliferating cell nuclear antigen) across 36 healthy tissue types and demonstrated that it significantly outperformed a majority of prognostic signatures developed for breast cancer (Supplementary Table 1) [27, 28]. Further highlighting the importance of proliferation rate, they determined that a majority of variation in breast cancer transcriptomes is correlated with proliferation and most random gene sets are significantly associated with breast cancer outcome due to their inherent relationship with a broad underlying proliferation signature [27, 28]. In our study, we examine the relative importance of proliferation to disease progression and patient prognosis across cancers using RNA-sequencing (RNA-seq) profiles from 19 cancers in 6,581 patients catalogued by The Cancer Genome Atlas (TCGA). We contrast these with 30 normal tissues from 8,553 patients from the Genotype-Tissue Expression (GTEx) Project to investigate proliferation indices across tissues types and disease stages (Supplementary Tables 2 and 3). We also demonstrate a strong relationship between tumor proliferation signatures and somatic mutation burden and identify genes containing single nucleotide variants associated with a proliferative phenotype across cancers. Finally, we provide on open-source R package, which calculates proliferation index based on gene expression and allows comparison of a proliferation-based model to models based on user-identified genes.

## RESULTS

### Proliferation index varies across tissues, cancer types, and tumor pathology

**Figure 1:**
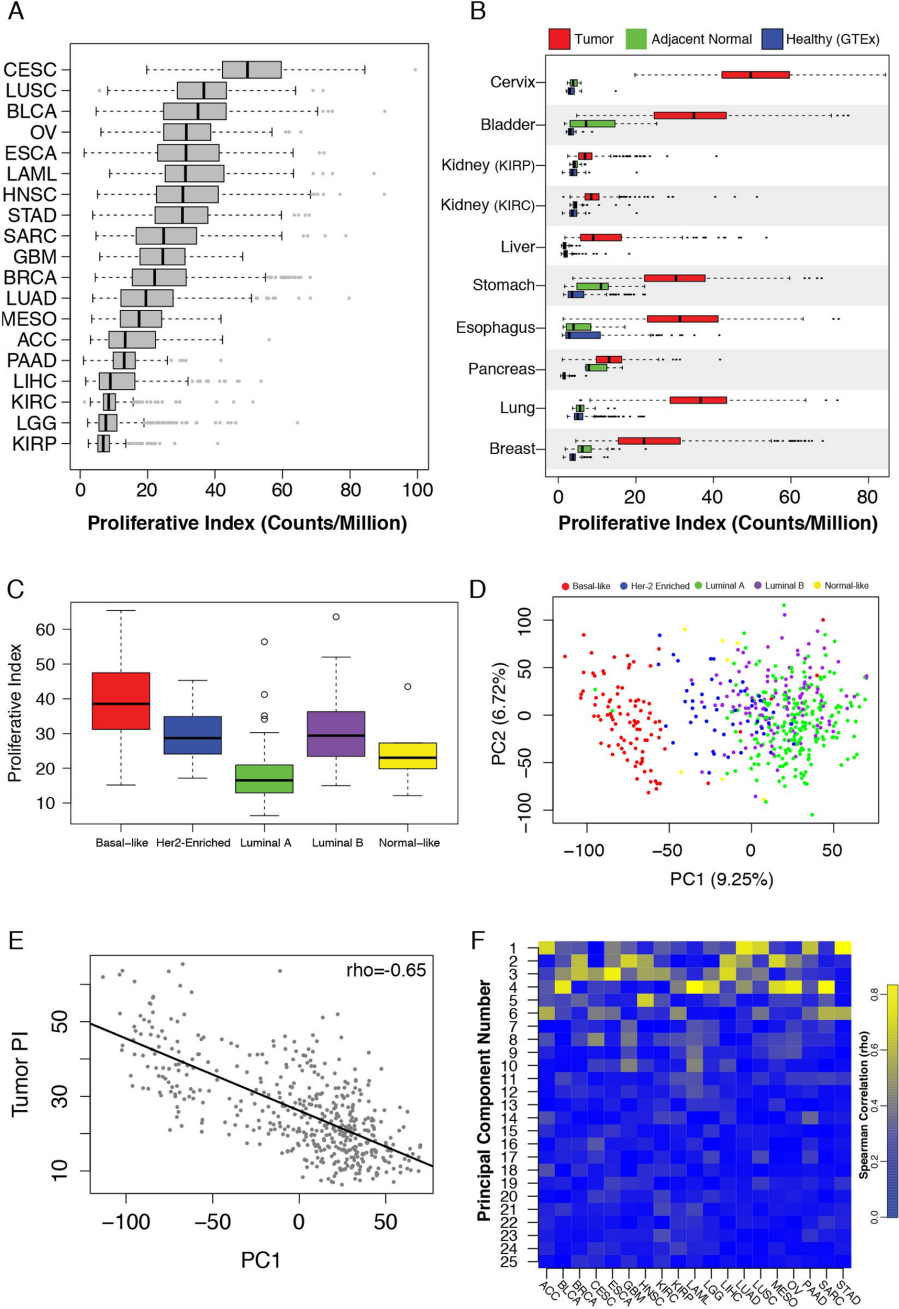
(**A**) Tumor proliferative index distributions across TCGA cancers. (**B**) Proliferative index values in healthy GTEx samples (blue), TCGA tumor-adjacent normal tissue (red) and TCGA tumor tissue (green). (**C**) Tumor proliferative index values across breast cancer PAM50 subtypes. (**D**) PCA of TCGA breast cancer samples stratifies tumors based on PAM50 subtypes. (**E**) The first principal component of the TCGA breast cancer data set correlates with tumor proliferative index. (**F**) Heatmap of principal component-tumor proliferation index correlations across cancers.

We compiled RNA-seq and associated clinical annotation data for 6,581 patients across cancers originating from 19 tissues. To be included in this study, clinical and RNA-seq data for a given cancer must have been available for at least 50 patients and at least 25 patients must have died from the disease to provide uncensored survival information. Examination of the proliferative index (PI), a measure of cell proliferation, within and across tumor types revealed a continuum of index values within each cancer and notable differences between cancers (Figure 1A). We compared tumor PI to previously compiled scores of tumor purity describing the proportion of non-cancerous cells within a sample across TCGA samples [29] as well as hematoxylin and eosin staining provided in clinical files associated with each sample. We found weak correlation with each metric (Spearman rank coefficient (rho) = 0.096 and −0.074) indicating that PI is largely independent of tumor purity estimates. An analysis of PI in healthy GTEx tissues revealed low PI values in post-mitotic tissues such as skeletal muscle and brain tissue and higher values in Epstein-Barr virus-transformed lymphocytes or tissues with high rates of cell turnover such as esophageal mucosa, vaginal epithelium and skin (Supplementary Figure 1). For every cancer with adjacent normal tissue available from TCGA (*n* = 12), the PI was higher in tumor tissue compared to adjacent normal tissue (Wilcoxon, *p* < 0.05). This was also true when comparing tumor tissue collected by TCGA to normal tissue collected from the same organs by the GTEx Consortium (*n* = 9), demonstrating tumorigenesis is accompanied by a characteristic increase in proliferation-related gene expression (Figure 1B).

**Figure 2:**
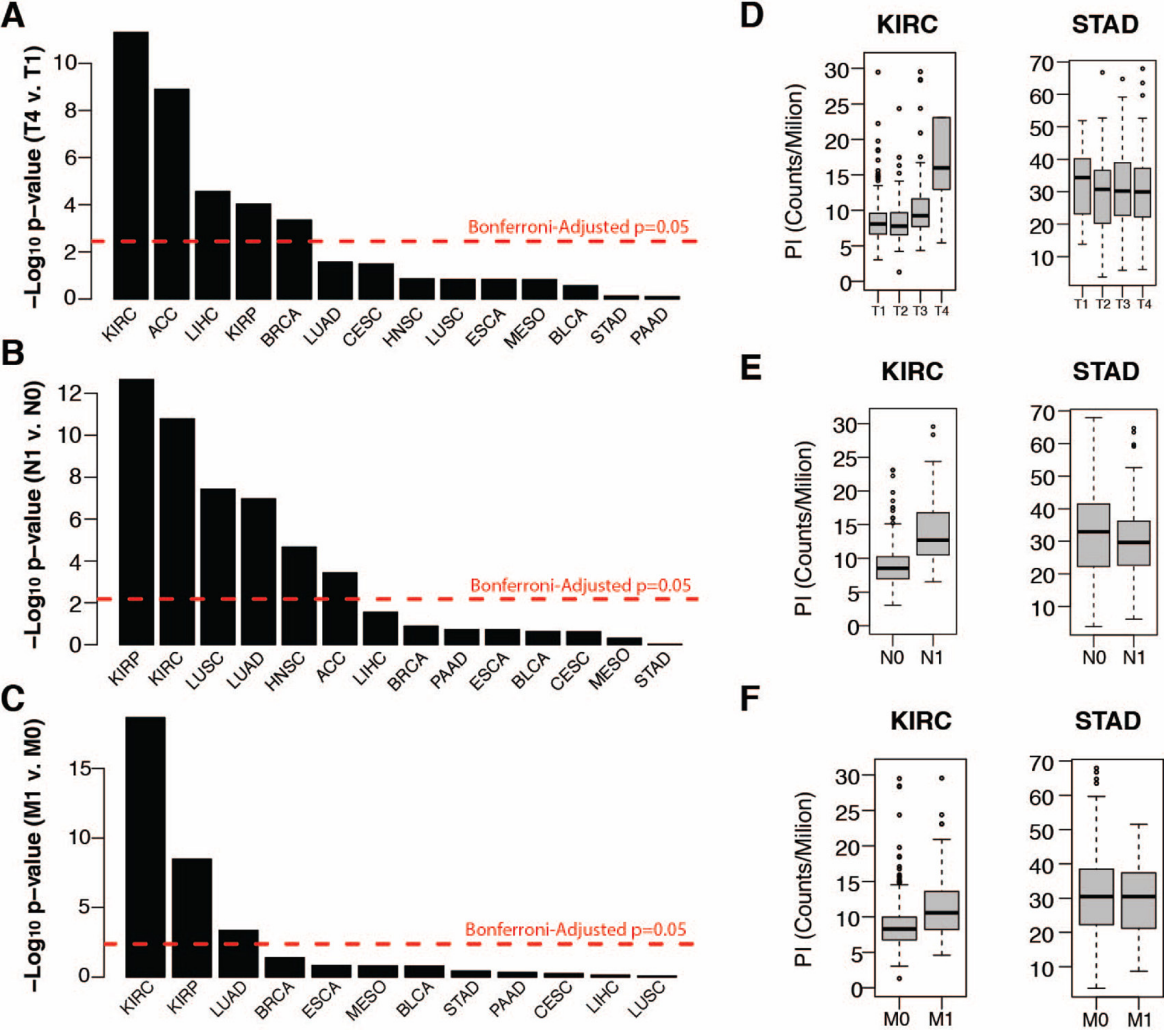
(**A**–**C**) Wilcox test negative log *p*-values of tumor proliferation comparisons between (A) tumor T stages 1 and 4, (B) tumor N stages 0 and 1 (nodal invasion), and tumor M stages 0 and 1 (metastasis) (C). (**D**–**F**) Distribution of tumor proliferation index across tumor T (D), N (E) and M stages for TCGA renal cell carcinoma (KIRC) and stomach adenocarcinoma (STAD).

The substantial size of the breast cancer cohort (*n* = 1,098) allowed us to investigate additional properties. Within breast cancer Prediction Analysis of Microarray 50 (PAM50) subtypes, PI values were highest among aggressive basal-like tumors and lowest among the less aggressive luminal A and normal-like subtypes (Figure 1C) [30]. Principal component analysis (PCA) of all gene expression levels in breast cancer confirmed that the first principal component (PC1) stratified subtypes (Figure 1D). Interestingly, PC1 was also strongly correlated with tumor PI (rho = 0.65) indicating that a large proportion of variance in breast cancer gene expression, including subtype delineations, is strongly associated with proliferation (Figure 1E). Moreover, examining PI across all cancers revealed strong correlations with early principal components in a majority of cancers, supporting previous observations that a large portion of variance across tumor transcriptomes is correlated with their proliferation index (Figure 1F). However, tumor PI was associated with pathologically assessed tumor stage, nodal invasion, and metastasis in only a subset of tumors analyzed, suggesting the importance of proliferation in tumor progression may vary considerably across cancers (Figure 2A–2C). PI values are plotted across each pathological grading characteristic for clear cell renal carcinoma (KIRC), a representative cancer for which PI is significantly associated with pathological stage, and stomach adenocarcinoma (STAD), a representative cancer for which PI is not associated with pathological stage (Figure 2D–2F).

### Cell proliferation is associated with overall survival in a subset of cancers

**Figure 3:**
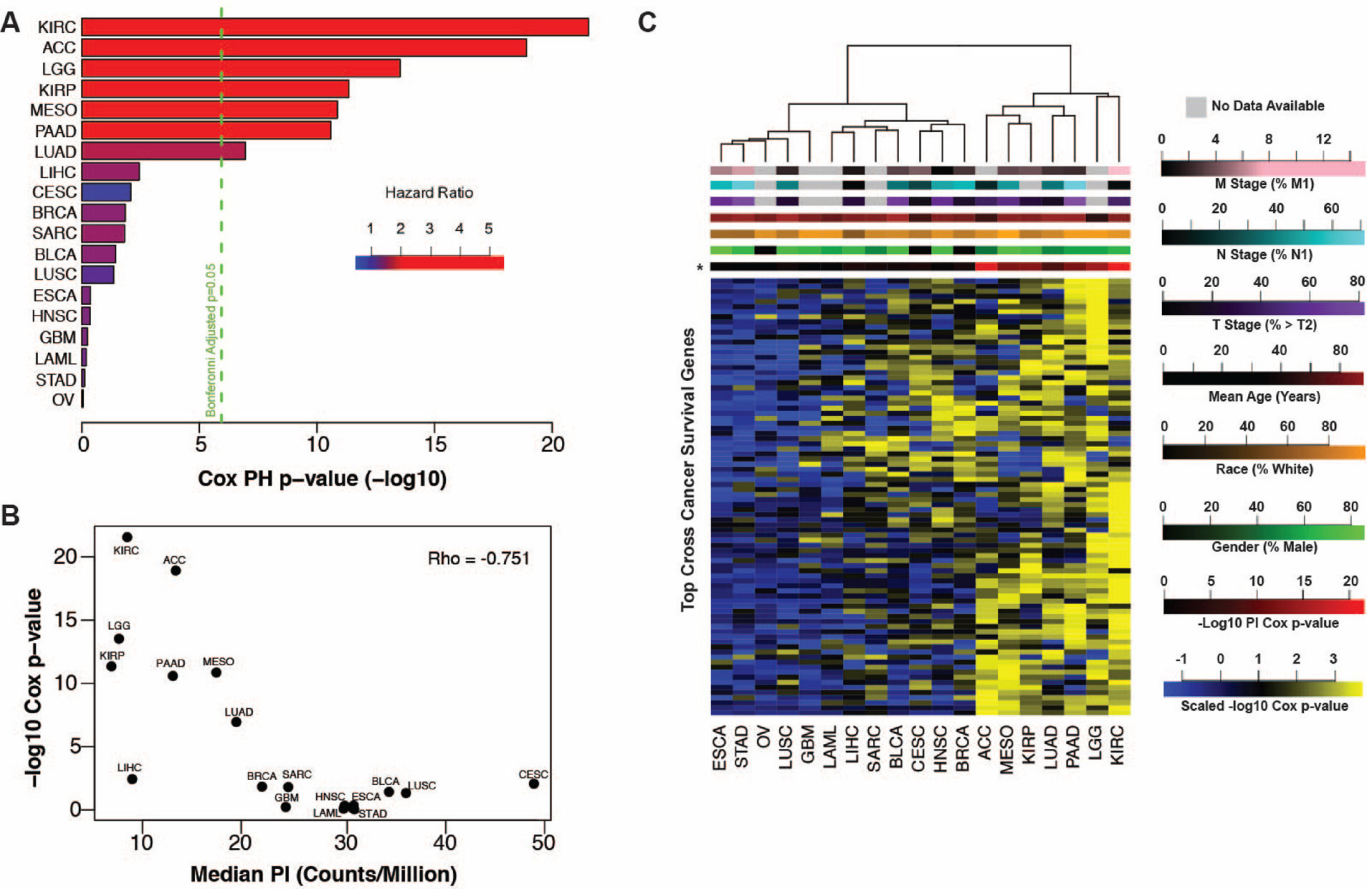
(**A**) Tumor proliferative index Cox regression negative log *p*-values plotted by cancer with the first seven cancers showing significant association with patient outcome. (**B**) Tumor proliferation index survival associations (Cox regression negative log *p*-values) are anti-correlated with the median tumor proliferation index of each cancer. (**C**) Heatmap of negative log Cox regression *p*-values of genes significant (*p* < 0.05, *n* = 84) in at least 9 of 19 cancers identifies PICs (right).

Next we assessed the relationship between tumor PI and patient survival. Cox proportional hazards models and Kaplan-Meier curve analysis revealed tumor PI was significantly associated with survival in a subset of cancers similar to those implicated in Figure 2 above (Figure 3A, Supplementary Figure 2). Strikingly, we found that cancers with the lowest PI had PIs more strongly associated with survival than cancers with a higher PI (Figure 3B). This may indicate that other tumor characteristics are more important to patient survival in cancers with the highest PIs. We tested this hypothesis by performing Cox proportional hazards regression on all transcripts in each cancer. Pathway analysis of transcripts significantly associated with survival confirmed an enrichment for proliferation-related gene ontology (GO) terms such as cell cycle, DNA replication, and cell division in cancers whose PI was associated with survival whereas other cancers showed a relative paucity of proliferation-related enrichment and favored cell metabolism, transport, reactive oxygen species response, angiogenesis and immune related terms (Supplementary Tables 4 and 5, Supplementary Figure 3).

No transcripts were associated with survival in all cancers, however 84 transcripts were associated with survival (Cox *p*-value < 0.05) in at least 9 of 19 cancers. Pathway analysis on these transcripts revealed enrichment for proliferation-related processes including mitosis, cell and nuclear division, and spindle formation (Supplementary Table 6). We clustered cancers by their respective Cox regression *p*-values for each of these 84 transcripts and observed two distinct clusters (Figure 3C). The first cluster, representing 12/19 cancers, has relatively few low *p*-values, indicating that survival patterns are relatively unique to each of these cancer types. The second cluster, consisting of the remaining 7 cancers, shows a much stronger enrichment for low *p*-values indicating a common, proliferation-related, survival phenotype. The second cluster of cancers, (which we refer to as proliferation-informative cancers, PICs), is identical to the subset of cancers for which the tumor PI was significantly associated with survival and is not enriched for any clinical or demographic parameter. Relaxing the threshold for the number of significant cancers required for a transcript to be included in the model did not significantly alter this clustering pattern (Supplementary Figure 4). To ensure this clustering pattern is not driven by general tumor or tissue expression patterns and is specific to survival associated expression relationships, we clustered individual patients based on the expression of the top 250 most variable transcripts across all cancers and were unable to recapitulate the previously observed PIC cluster (Supplementary Figure 5).

**Figure 4:**
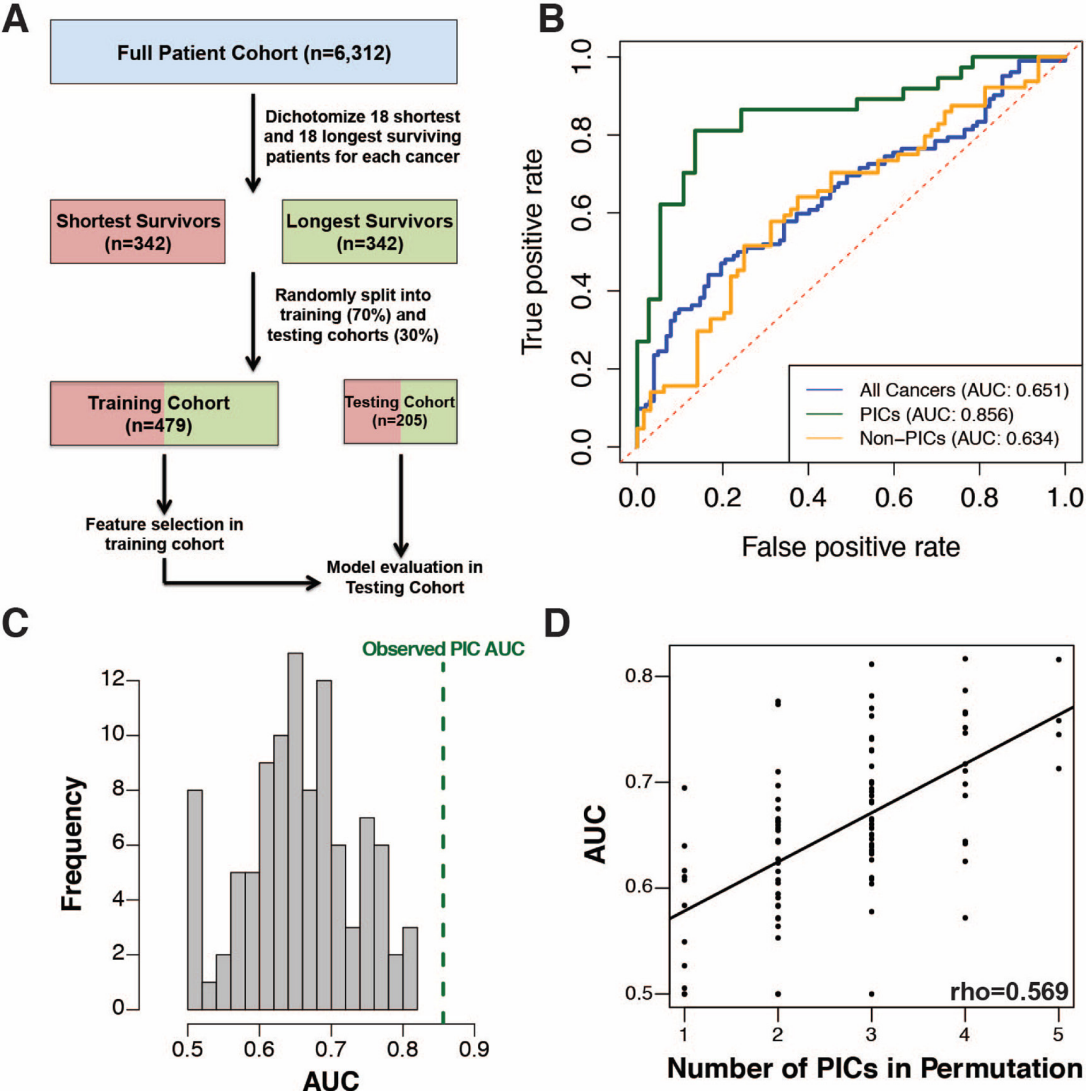
(**A**) Workflow for cross-cancer survival model generation. (**B**) ROC curve for multivariate Cox regression with LASSO for variable selection on all 19 cancers (blue), PICs only (green) and non-PICs only (orange). (**C**) Histogram showing the distribution of ROC curve AUC values for survival models generated on 100 randomly sampled sets of cancers equivalent in number to the PICs. (**D**) The ROC curve AUC values are directly proportional to the number of PICs included in random sample sets.

To further investigate cancer survival patterns, we sought to develop a cross-cancer prognostic model using the expression level of all genes as potential predictive features by selecting an equivalent number of the shortest surviving and longest surviving patients from each cancer type and randomly partitioning all samples into training and testing cohorts for model development and evaluation (Figure 4A). A multivariate Cox regression model with L1-penalized log partial likelihood (LASSO) for feature selection had relatively poor performance (receiver operating characteristic area under the curve, ROC-AUC = 0.651) when trained on the full set of cancers, however when limited to just PICs, performance improved significantly (ROC-AUC=0.856, *p*-value = 0.0004, Supplementary Tables 7 and 8). This again demonstrates PICs share a common survival signature (Figure 4B, Supplementary Table 9). To assess the uniqueness of the PICs' model performance, we randomly selected 1000 sets of 7 cancers for model training and none demonstrated the performance achieved by the PIC-only model (Figure 4C). In fact, model performance across our permutations was strongly correlated with the number of PICs incorporated into each model (Figure 4D). This trend was also observed using other predictive modeling approaches (Supplementary Figure 6). To assess whether our PIC model could perform well as a continuous metric of survival outside of our pre-dichotomized cohort, we applied it to the full patient cohorts for each PIC. In all PICs, model prediction values were successful at stratifying patients by prognosis (Supplementary Figure 7). To facilitate PI exploration, we have developed an R package (available at github https://github.com/blasseigne/ProliferativeIndex and on CRAN, DOI: 10.5281/zenodo.400951), ‘ProliferativeIndex’, which calculates and analyzes PI across a user's tumor RNA-seq dataset and compares the PI's prognostic performance with a user's survival model.

### Linking proliferation index and drug sensitivity

Many chemotherapies target proliferation-associated processes, therefore we hypothesized that sensitivity to these drugs may be correlated with PI. We took advantage of two public data sets to address this question. The Cancer Cell Line Encyclopedia [31] provides gene expression and drug sensitivity data for a panel of cancer cell lines and the Connectivity Map project [32] provides gene expression data following drug treatments in cancer cell lines. While there are significant caveats to using this data, namely the applicability of a tissue-derived index in an *in vitro* culture environment, an analysis of these correlations could provide testable hypotheses about drug sensitivity. We calculated correlations between the proliferative index and therapeutic response using two orthogonal cancer cell line datasets [31, 32] and found that irinotecan, topotecan, panobinostat and paclitaxel showed a significant correlation between EC50 concentrations and PI (Supplementary Figure 8A). Using the connectivity map data, we confirmed the expected result that in MCF7, a breast cancer cell line, estradiol, a known activator of cell proliferation in ER positive breast cancers is ranked in the top 20% of drugs investigated that correlate with PI [32]. In agreement with our finding that response to the HDAC inhibitor paribinostat is correlated with PI, we found that treatment with HDAC inhibitors in the CMap database (vorinostat and trichostatin A1) rank in the bottom 10th percentile of all drugs tested (Supplementary Figure 8B–8C) indicating that they reduce growth.

### Proliferation and somatic mutation burden

**Figure 5:**
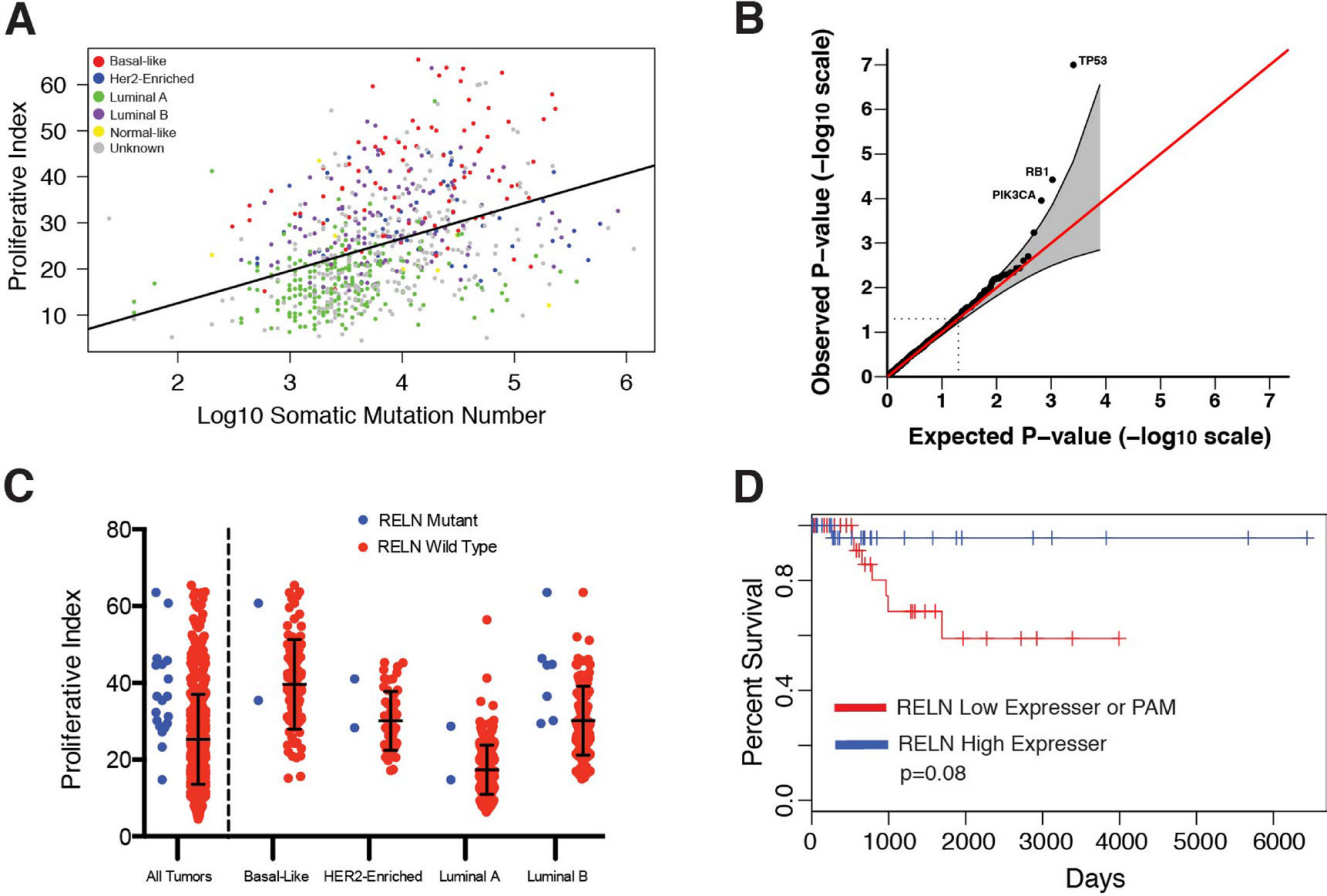
(**A**) Tumor proliferative index is correlated with TCGA breast cancer somatic mutation burden. (**B**) Q-Q plot of *p*-values derived from gene mutation burden-proliferative index associations. (**C**) TCGA breast tumors containing non-synonymous mutations in *RELN* have higher proliferative index compared to wild-type. (**D**) Kaplan-Meier survival plot shows reduced expression or protein-altering mutations in RELN are markers of poor prognosis in patients with basal breast cancer.

Increased rates of cell division, particularly in cancer cells whose repair mechanisms are diminished, might be expected to correlate with mutation burden. We assessed the relationship between tumor proliferation and somatic mutation burden in tumor exomes generated by TCGA and previously analyzed by Kandoth et al. [33]. We found a strong correlation between tumor PI and the number of somatic mutations both across multiple cancers and within each cancer (Supplementary Table 10). Notably, total mutation burden and PI were most strongly associated in breast cancer (rho = 0.45, Figure 5A). Correlations were also strong within each breast cancer subtype (rho>0.3) except for Her2-enriched tumors (rho<0.025). We next examined genes whose single nucleotide variation (SNV) burden most strongly associated with proliferation and found three well-established cancer driver genes (*TP53*, *RB1*, and *PI3K*) consistently implicated across cancers (FDR<0.1, Figure 5B and Supplementary Table 11). Apart from these top driver genes, mutations associated with proliferation are tumor-specific. For example, *RELN* was among the top 5 genes in breast cancer ranked by protein altering mutations associated with increased PI values in each subtype (Figure 5C). Breast cancer patients within the basal-like subtype tended to have shorter survival times if their tumors harbored protein altering mutations or were low expressers of *RELN* compared to patients with tumors expressing *RELN* at high levels (*p* = 0.08, Figure 5D).

## DISCUSSION

We have described an RNA-seq based analysis of cell proliferation across 19 cancers in 6,581 patients. We show a high degree of variability in the relative expression of proliferation-associated genes both within the same cancer type and across different cancers. Interestingly, cancers with relatively low expression of proliferation-associated genes tended to be those for which PI was strongly associated with pathology-based markers of tumor staging and survival. This suggests that some cancer types may saturate their capacity for proliferation at early stages, so other factors such as invasion, immune suppression, and drug transport to are more important for patient prognosis. Our data suggest proliferation may play a more prominent role in dictating prognosis in cancers that avoid maximal rates of cell division during early tumorigenesis and possess relatively lower absolute levels of proliferation-associated expression. Future studies investigating evolutionary histories of tumors could investigate this phenomenon in more detail, as there may be considerable heterogeneity between cancers in the genes important to predicting patient survival and these studies could inform the use of targeted therapies to cancer-specific pathways most relevant to patient outcomes. Using existing data, we demonstrated that PI is significantly correlated with the sensitivity to a subset of drugs *in vitro*. Important to consider, however is that in making these assessments, we used gene expression measurements taken from cell lines, which are cultured in dramatically simpler environments and may exhibit different growth patterns than tumor cells. Furthermore, cell numbers were primarily obtained by quantifying the amount of ATP per well, which could be confounded by alterations in cell metabolism. This analysis provides new hypotheses about therapeutic efficacy and future studies are necessary to confirm the relevance of these observations at physiologically constrained *in vivo doses*.

Somewhat surprisingly, breast cancer was not one of the cancers exhibiting the strongest association between patient survival and proliferation index despite several previous studies to the contrary [18–20, 27]. Based on these studies it seems clear that the proliferation is associated with breast cancer prognosis. In our study, the power to make prognostic observations in the TCGA breast cancer cohort is limited by the fact that the cohort has been followed for a relatively short amount time so greater than 90% of the cohort was still alive at the time of analysis. Survival times and PI are also linked to breast cancer subtype (Supplementary Figure 9), thus the subtype representation of a cohort could strongly influence the prognostic utility of patient PI.

Additionally, we demonstrated that survival-associated gene expression patterns are not common across all cancers. However, a subset of cancers (PICs) share an overlapping signature enriched for proliferation-associated genes. We developed a common prognostic signature that contains several genes previously implicated in cancer prognosis and that accurately predicts patient survival across all seven PICs. For example, *CKS2* is a regulatory protein that binds the catalytic subunit of cyclin-dependent kinases and is essential for kinase function in regulating the cell cycle [34, 35]. *CRYL1* has been shown to regulate G_2_-M phase transition and expression has been linked to patient prognosis [36]. *DNA2* is a DNA helicase that plays an important role in processing Okazaki fragments during DNA replication and *DNA2* expression is correlated with patient survival [37]. *HJURP* is a histone chaperone shown to play a role in the progression of gliomas and breast tumors [38, 39]. *SUOX* had the largest absolute coefficient in our model; however, its role in cancer progression is less clear. It is a mitochondrial enzyme that catalyzes the conversion of sulfite to sulfate and has been described in one study as a prognostic immunohistochemical marker for hepatocellular carcinoma [40], yet its functional role and importance in cancer remains unclear. Future prognostic modeling within PICs or cross-cancer modeling that includes PICs should consider the significant role of tumor proliferation-associated expression before interpreting biological mechanisms for prognosis-associated genes. Additionally, newly developed prognostic models in PICs should outperform general transcriptome associations with survival before mechanistic interpretations are made.

Proliferating tumors, which must constantly replicate their genomes, are prone to increased mutation rates, a phenomenon consistent with our finding that tumor PI is strongly correlated with somatic mutation burden both within and across cancers. This may provide a potential mechanism by which increased proliferation rates associate with poor outcomes as increasing the mutational heterogeneity of a tumor may lead to avenues of escape from targeted drug therapies [41]. However, we did not see a strong relationship between tumor mutation burden-PI correlation strength and PI's prognostic ability across cancer types. In fact, breast cancer, the cancer type with the highest mutation burden-PI correlation, was not designated a PIC in our study. Further comparisons of gene mutation burden with tumor PI revealed three well-known tumor suppressor genes (*TP53*, *RB1*, and *PI3K*) to be significantly associated with proliferation across multiple cancers, consistent with large bodies of previous work. For example, a large analysis of *TP53* levels in node-negative breast cancer revealed decreases in *TP53* were strongly associated with a concurrent increase in both tumor proliferation and poorer patient outcomes [42]. Moreover, an extensive body of literature supports the fact that *PI3K*'s ability to upregulate proliferation machinery through downstream activation of the *AKT/mTOR* pathway [24]. Focusing on breast cancer, the largest cancer cohort available, we found one relatively less investigated gene, *RELN*, among the top PI associated genes. We found that protein-altering mutations in *RELN* are associated with increased tumor PI in each breast cancer subtype, and that low levels of *RELN* expression are associated with poor prognosis within the basal subtype. Decreased expression and epigenetic silencing of *RELN* has previously been associated with advanced stage and poor prognosis in several cancers [43–47] and recent work has shown that loss of RAS signaling by disrupting interactions with PI3K increases extracellular RELN levels, resulting in decreased tumor aggressiveness via activation of cell adhesion pathways [48]. Our findings indicate there may be intriguing roles for *RELN* in the progression of breast cancer particularly related to tumor proliferation; however, future functional investigations are necessary to confirm its role.

An important limitation to this study is its reliance on a relatively simplistic model for estimated tumor proliferation rates – namely the expression of a group of genes strongly associated with proliferation across healthy tissues. Future work investigating expression patterns associated with more precise measurements of tumor proliferation is essential to expanding upon this analysis. Furthermore, the relationships previously described, particularly in regards to identifying PICs, should be further investigated in future large-scale, multi-cancer expression studies because TCGA is currently the only resource of sufficient scale.

In conclusion, our study provides a comprehensive characterization of tumor proliferation rates and their association with disease progression and prognosis across cancer types and highlights specific cancers that may be particularly susceptible to improved targeting of proliferation-related gene pathways. We have expanded upon previous work developing a generalizable proliferation related-classification framework and provided a community-available resource to investigate further the role of proliferation both within and between cancers.

## MATERIALS AND METHODS

### TCGA and GTEx data acquisition

RNA-seq and associated patient clinical data were obtained from the TCGA data portal (tcga-data.nci.nih.gov) in June 2015. (Supplementary Table 1) Level 3 RNASeqV2 raw count data was used for downstream analysis. This included quantification of > 20,000 transcripts. Relevant clinical information for each patient was obtained from the associated “clinical_patient” and “clinical_follow_up” files, with survival time calculated as the maximum “days_to_death” or “days_to_last_followup” column value from the “clinical_patient” file or any “clinical_follow_up” file. All staging information was obtained from the “pathologic_T”, “pathologic_N”, and “pathologic_M” columns in the “clinical_patient” file. GTEx (gtexportal.org) V6 RNA-seq data for all available tissues was obtained in January 2016 (Supplementary Table 2). This data included quantification of > 40,000 transcripts.

### All analysis was performed using R [49] (Version 3.2.1) with RStudio [50] (Version 0.99.891)

#### Data normalization and PI calculation

The PI was calculated as previously described by Venet et al. [27]. Briefly, a sample's PI was defined as the median expression level of the original 131 genes found to be most associated with PCNA expression across 36 tissue types. For cross-cancer or cross-tissue comparisons, raw read counts were normalized to counts-per-million (CPM) prior to PI calculation. For intra-cancer analyses, raw counts were variance stabilized using the ‘DESeq2′[51] (Version 1.8.2) package function “varianceStabilizingTransformation” prior to PI calculation or survival analysis.

### PI comparisons and survival association analysis

All cross-sample PI comparisons were conducted with two-sided Wilcox tests via the base ‘stats’[49] (version 3.2.1) package wilcox.test function. PI-survival associations were determined using ‘survival’ [52, 53] (version 2.38-3) and ‘survcomp’ [54, 55] (version 1.18.0) packages. Cox regressions were performed with the coxph function to regress overall patient survival on PI and Wald test *p*-values were reported. Kaplan-Meier curves were generated for tumors in the top and bottom quartiles of PI using the survfit function and significant differences between survival curves were assessed with the survdiff function. Dendrograms of cancer clustering based on negative log_10_ Cox regression *p*-values were constructed with the hclust function using Ward clustering. A heatmap of cross-cancer survival associated genes (uncorrected *p*-value < 0.05 for at least 9/19 cancers) was generated on negative log_10_ Cox regression *p*-values generated for each transcript measured in TCGA Level 3 data. Models that failed to converge, based on previously established criteria employed by the ‘survival’ package (almost always due to a maximum likelihood estimate of a coefficient nearing infinity) [52], were assigned a *p*-value of 1. The heatmap was generated with the R gplots [56] (version 2.17.0) heatmap.2 function using Euclidean distance measurement and Ward clustering.

### Pathway analysis

Pathway analysis was conducted on the 162 cross-cancer survival associated genes with uncorrected Cox *p*-values < 0.05 across all PICs using the Database for Annotation, Visualization and Integrated Discovery (DAVID, v6.7) [57, 58] pathway analysis with default settings. All unique gene names available in the TCGA Level 3 count data were used as a background for analysis. Gene ontology enrichment analysis of expression-survival associations in each cancer was conducted with GOrilla (http://cbl-gorilla.cs.technion.ac.il) in “single ranked list of genes” mode. GO terms were condensed into broader categories for visualization with REVIGO (http://revigo.irb.hr) [59].

### Cross-cancer survival model

Variance stabilized transcript count data was scaled within each cancer prior to combining cohorts for all cross-cancer survival model generation. For each cancer, the 18 shortest surviving patients who succumbed to disease and the 18 longest surviving patients were identified for initial analyses. Only 18 patients were selected because this represented the top and bottom quartiles of the mesothelioma cohort, the smallest cohort included in this study. Patients were indexed under an “outcome” variable as “1” if they were in the longest surviving cohort and “0” if they were in the shortest surviving cohort. We then generated two models for predicting patient outcome from tumor gene expression using the basic formula:

### Outcome ~ expression

where “Outcome” is the patient prognosis as described above and “Expression” represents the scaled expression level of all genes included in the TCGA tier 3 analysis. The first model trained included all 19 cancers and the second included only PIC cancers (KIRC, ACC, LGG, KIRP, MESO, PAAD, and LUAD). PICs were defined as cancers with Bonferroni-corrected PI Cox regression *p*-value of less than 0.05, which are also the cancers who clustered together when considering only the cross-cancer significant survival transcripts as described above. Prior to model training, the ‘caret’[60] (version 6.0–64) createDataPartition function was used to split the full cross-cancer and PIC-only data sets into a training cohort containing 70% of patients and a testing cohort containing 30% of patients, while conserving a roughly equivalent number of shortest and longest overall survival patients within each partition. Models were trained without knowledge of the proliferation index with all genes capable of acting as features in the training cohort.

### LASSO

A LASSO regression model was trained on the full cross-cancer and PIC and non-PIC only training cohorts using the glmnet [61] (version 2.0–2) cv.glmnet function with regression family set to “binomial” and nfolds set at 5. This generated a binomial regression model, which used a lambda penalty optimized using 5-fold cross validation within the training cohort. The optimal lambda penalty was defined as the smallest model with a cross validation mean squared error within one standard deviation from the minimum value.

### Ridge

A ridge regression model was also trained with the cv.glmnet function with identical parameters as the LASSO model described above, except the alpha parameter was set to 0.

### Random forest

A random forest model was trained on the full cross-cancer and PIC only cohorts using the randomForest [62] (version 4.6–12) package. Models were generated with the randomForest function using default settings except mtry was limited to 1000.

### SVM

A linear support vector machine model was trained on the full cross-cancer and PIC only cohorts using the e1071 [63] (version 1.6–7) package. The model was trained using the svm function with kernel set to “linear” and “cross” set to 5. The cost parameter was optimized for each cohort by finding the value that minimized the 5-fold cross validation squared error within the training cohort after trying a series of values ranging from 0.00001 to 10000.

### Model evaluation

Performance was evaluated for each model by test set ROC curve AUC generated by predictions made on the testing cohort using the predict function and the ROCR[64] package (version 1.0–7).

### Permutation

The significance of model performance in the PIC only cohort for each machine learning approach was assessed by randomly sampling seven cancers, dichotomizing the cohorts, training each model in an identical manner as described above for the PIC only cohort, and comparing ROC AUC curves for each resulting random sample. We used the webtool http://vassarstats.net/roc_comp.html to show a significant improvement in AUC for the PICs.

### Full cohort performance assessment

The LASSO model derived from the PIC-only cohort was applied to the full patient cohorts of each individual PIC to assess performance in a non-dichotomized setting. LGG, KIRC, and LUAD had greater than 25 uncensored patients remaining after removing patients in the training set, so for these cancers the model was applied only on patients that were not used to train the original model. Because KIRP, PAAD, MESO, and ACC had a limited number of remaining patients, the PIC LASSO model was applied to the full cohort including patients that were used to train the original model. The top and bottom quartiles of predicted survival were compared using Kaplan-Meier curves as described above.

### Drug associations with proliferation index

To correlate sample PI with drug efficacy, EC50 values for 24 drugs and normalized microarray expression data for 486 cancer cell lines was obtained from the Cancer Cell Line Encyclopedia [31]. The specific files used for analysis were “CCLE_NP24.2009_Drug_data_2015.02.24.csv” and “CCLE_Expression_2012009-29.res” (downloaded in June 2016). Proliferation index was calculated in a similar manner as described above by taking the median normalized expression value for each probe set mapping to a gene contained within the proliferation index. To measure impact of drug treatment on PI, expression profile data of MCF7 cells treated with 1309 drugs and their corresponding vehicle controls was obtained from the Connectivity Map data set [32]. The “rankMatrix.txt” file (downloaded in June 2016) was used for downstream analysis. This file consists of a probe set by treatment matrix with each probe set given a ranking (from 1 to the total number of probes – 22,777) corresponding to the magnitude of differential expression of that probe set after treatment with a drug relative to its vehicle control with a ranking of 1 assigned to the highest positive change in expression and 22,777 assigned to the lowest negative change in expression. The relative impact on PI of different treatments was compared by calculating a median ranking for all probe sets mapping to genes used in the calculation of PI for each treatment and subsequently ranking drugs according to the percentage of drugs with a higher PI ranking. Cumulative distribution functions of all PI-probe set rankings for drugs identified by the CCLE analysis were compared using a Kolmogorov-Smirnov test.

### Breast cancer subtyping

Subtype assignments for patients in the BRCA cohort were obtained from a previous TCGA analysis of breast cancer [65]. The “PAM50 mRNA” column in Supplementary Table 1 was used for those patients who met our criteria for analysis. Principal component analysis was performed using the prcomp function on the BRCA cohort on all variance stabilized transcript data.

### SNV-point mutation analysis

Somatic mutations were obtained from Kandoth, et al. [33] for 12 TCGA ‘Pan-Cancer’ datasets. We found 2,336 patients that overlapped from 9 cancers with the TCGA gene expression dataset and obtained somatic mutations for those patients from Kandoth, et al.'s Supplementary Table 2 where the authors used common, stringent filters to ‘ensure high quality mutation calls’ across those samples. Correlations between tumor PI and somatic mutation burden were calculated by calculating a Spearman correlation between the log_10_ of the sum of all mutations identified for each patient and the patient PI both across and within each cancer type. To identify genes with mutation status associated with PI, we performed Wilcoxon rank tests of PI between tumors containing a missense or nonsense mutation and tumors containing synonymous or no mutation for each gene with at least 5 mutations present in each cancer. This analysis was not performed on cancers with less than 100 genes meeting these criteria (*n* = 3). To identify significant cross-cancer trends, we used Fisher's combined *p*-value method on each gene mutated at least 5 times in at least 2 cancers.

## SUPPLEMENTARY FIGURES AND TABLES









## Abbreviations

MAPK: mitogen activated protein kinase
PI3K: phosphoinositide 3-kinase
PCNA: proliferating cell nuclear antigen
RNA-seq: RNA-sequencing
TCGA: The Cancer Genome Atlas
GTEx: Genotype-Tissue Expression
PI: proliferative index
PCA: principal component analysis
KIRC: clear cell renal carcinoma
STAD: stomach adenocarcinoma
LGG: low grade glioma
BRCA: breast adenocarcinoma
STAD: stomach adenocarcinoma
PAAD: pancreatic ductal adenocarcinoma
ACC: adrenocortical carcinoma
GO: gene ontology
PICs: proliferation-informative cancers
HDAC: histone deacetylase
CMap: Connectivity Map Project
