# Supplementary material for: RNA sequencing-based cell proliferation analysis across 19 cancers identifies a subset of proliferation-informative cancers with a common survival signature
Source: Oncotarget. 2017 Apr 8;8(24):38668–81. doi: 10.18632/oncotarget.16961 (PMC5503562; doi:10.18632/oncotarget.16961)
Supplement: Supplementary file 1 [file oncotarget-08-38668-s001.pdf]

# RNA sequencing-based cell proliferation analysis across 19 cancers identifies a subset of proliferation-informative cancers with a common survival signature

## Supplementary Materials

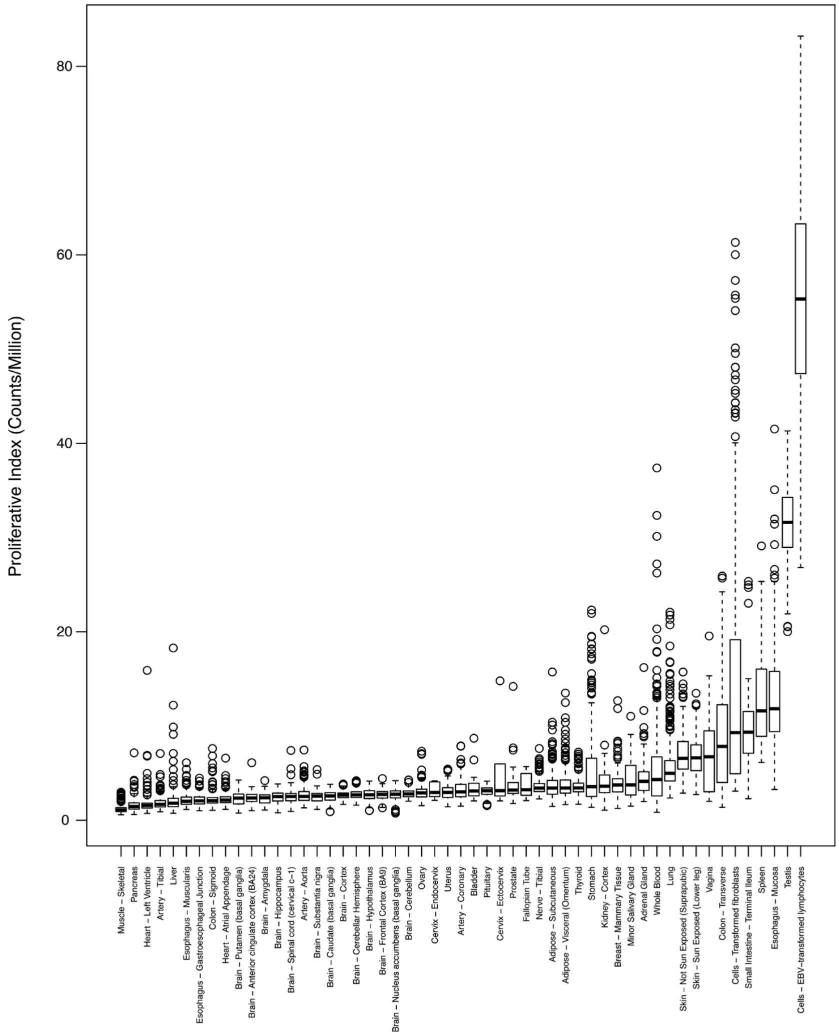


**Supplementary Figure 1: Proliferative index distributions across GTEx healthy tissues.**

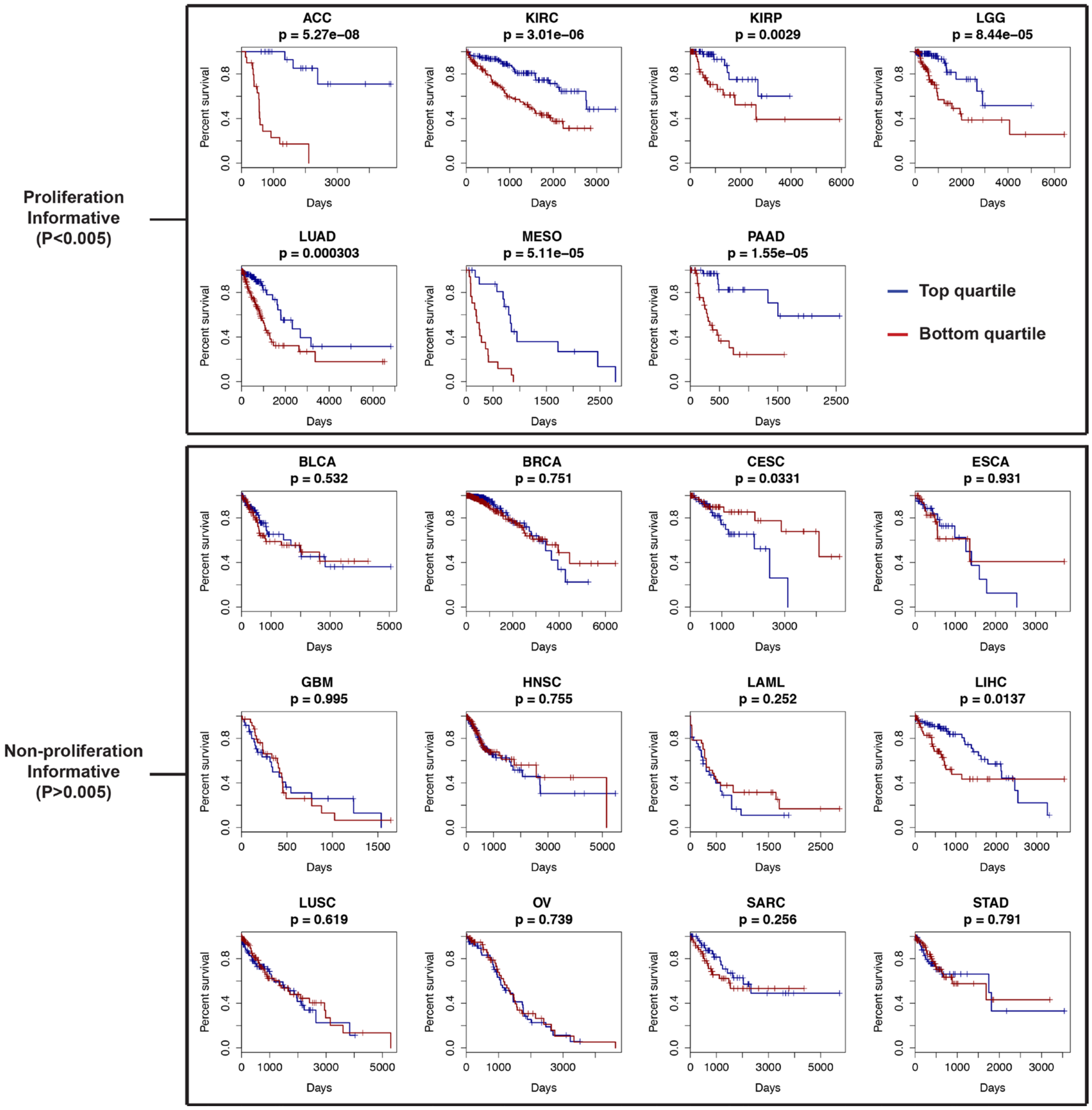


**Supplementary Figure 2: Kaplan-Meier curves for the top and bottom quartiles of tumor proliferative index across TCGA cancers.**

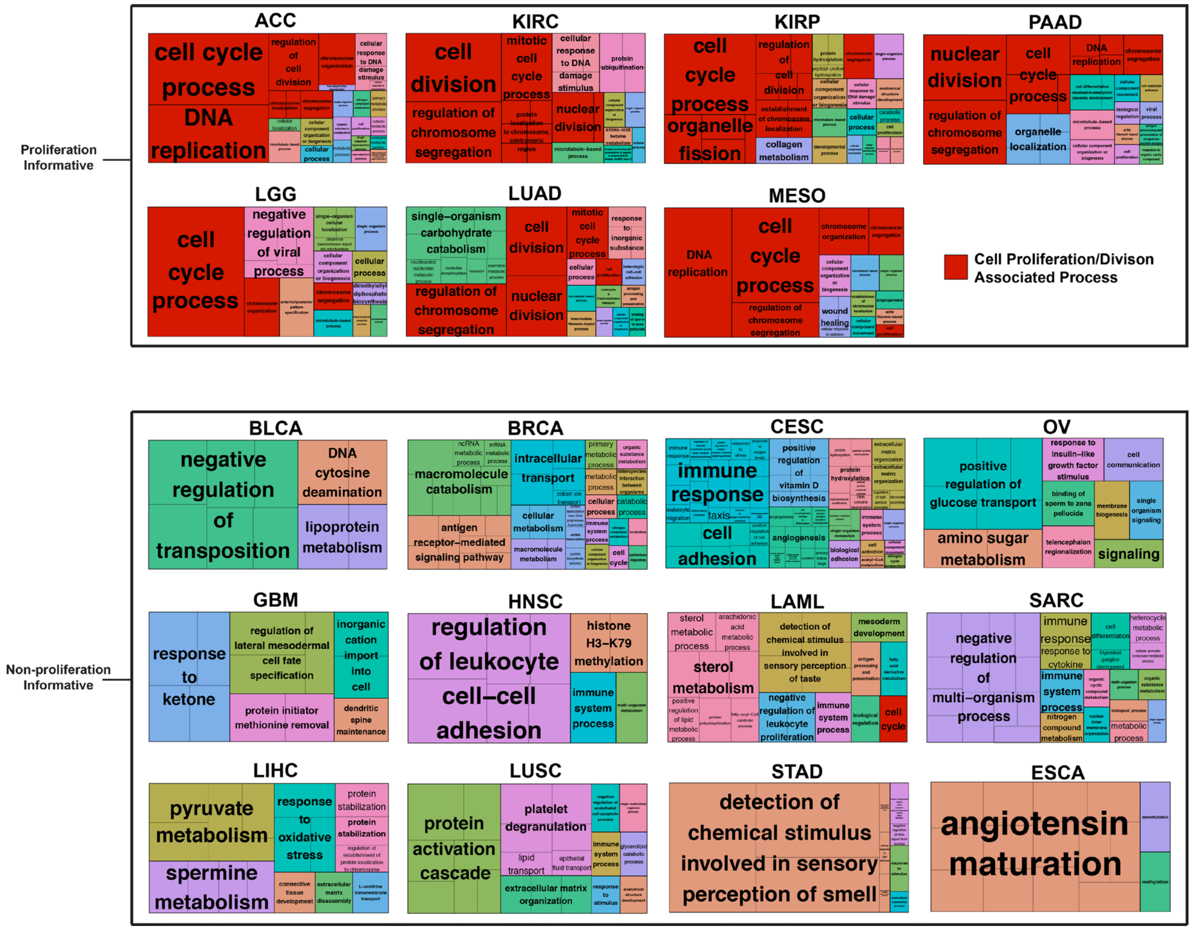


**Supplementary Figure 3: Gene ontology enrichment analysis on survival associated genes in each TCGA cancer.** Cell proliferation and division associated modules are colored in red. Module size is proportional to the number of significant genes contained within the module.

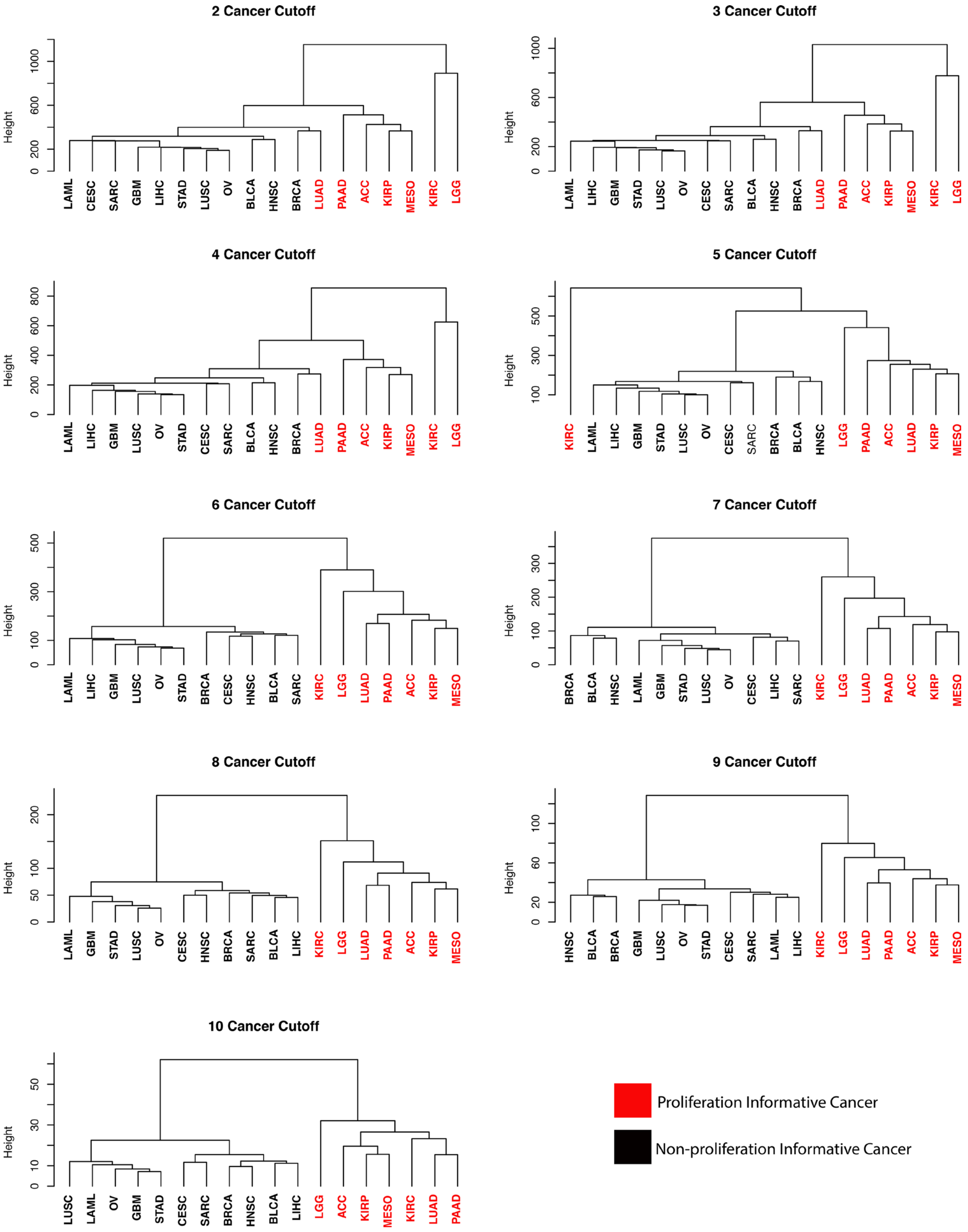


**Supplementary Figure 4: Dendrograms showing cancer clustering based on survival associated *p*-values using a sliding cutoff for inclusion of genes.** Gene inclusion cutoff ranged from requiring genes to be significant (Cox $p < 0.05$) in at least 2 cancers to at least 10 cancers. The PIC clustering pattern in Figure 3 is maintained across cutoffs.

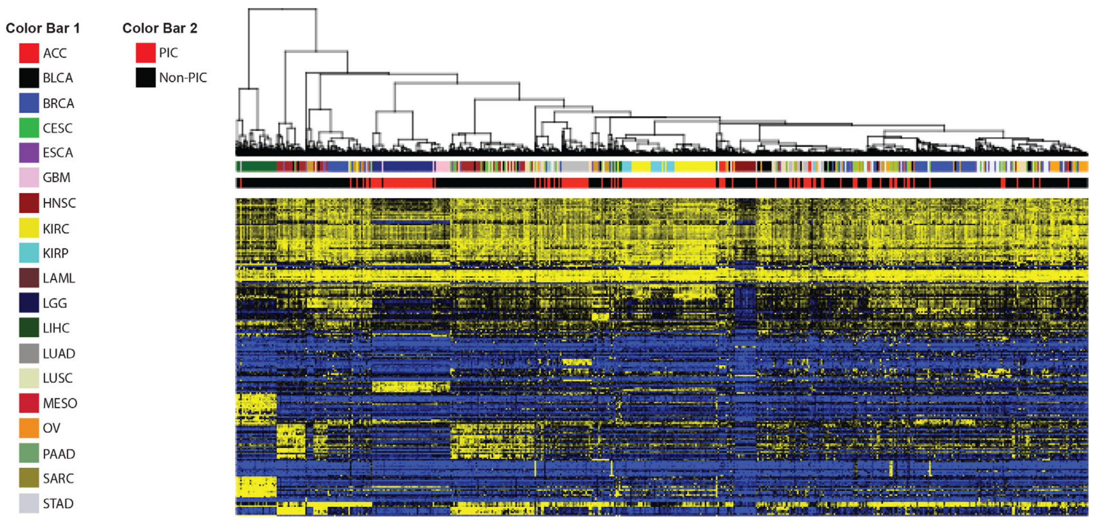


**Supplementary Figure 5: Tumor sample clustering based on expression levels of the top 250 most variable genes across all TCGA samples included in our analysis.** Patients with the same cancers tend to cluster together despite PIC status.

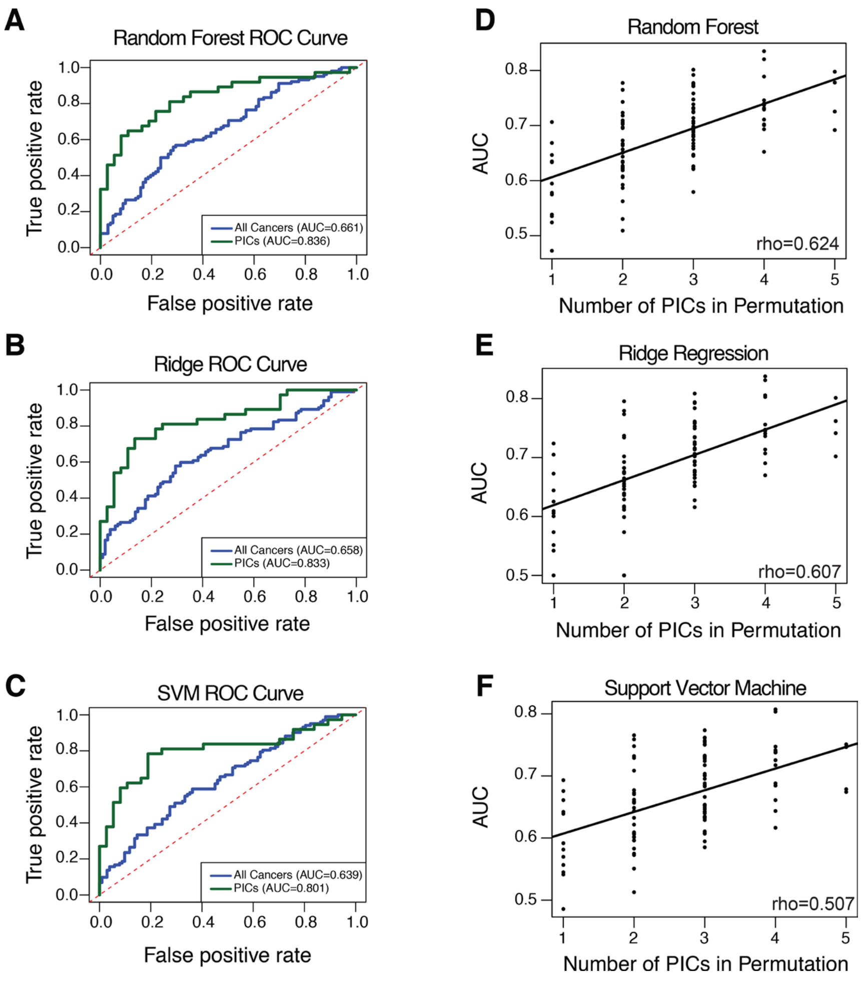


**Supplementary Figure 6:** Cross-cancer survival model performance generated on all cancers (blue) and PICs (green) with random forest (**A**), ridge regression (**B**), and support vector machines (**C**). (**D**–**F**) ROC curve AUC values from survival models generated on random sets of cancers equivalent in number to PICs are directly correlated with the number of PICs included in the random sample for each survival modeling approach.

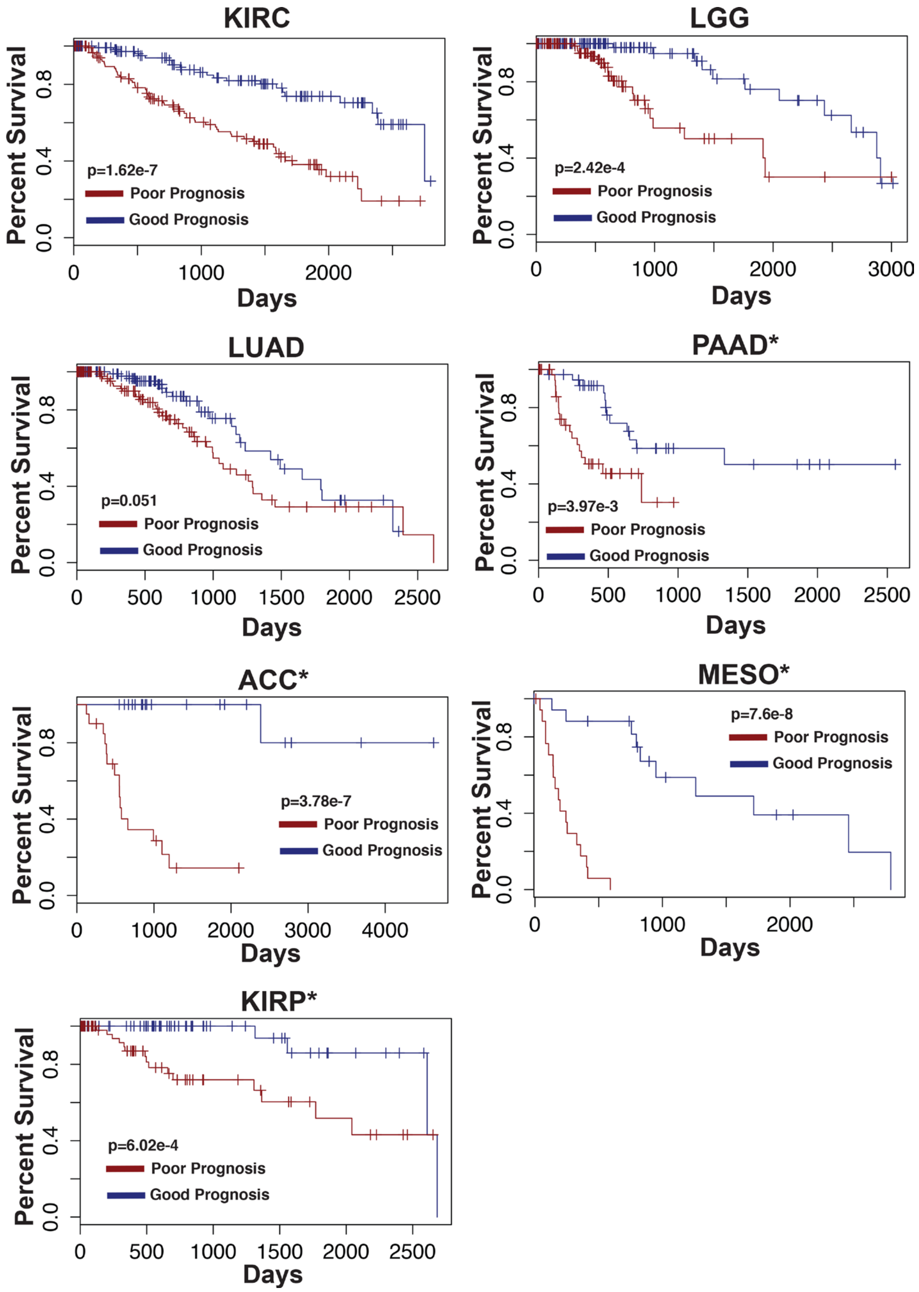


**Supplementary Figure 7: PIC LASSO based cross-cancer survival model performance on the full cohorts for each PIC.** Kaplan-Meier curves represent the top and bottom quartiles of patients in terms of predicted prognosis. PAAD, ACC, MESO, and KIRP had an insufficient (< 25) number of patients remaining after removing patients used to train the model, thus those Kaplan Meier curves include patients used in the initial training set.random sample for each survival modeling approach.

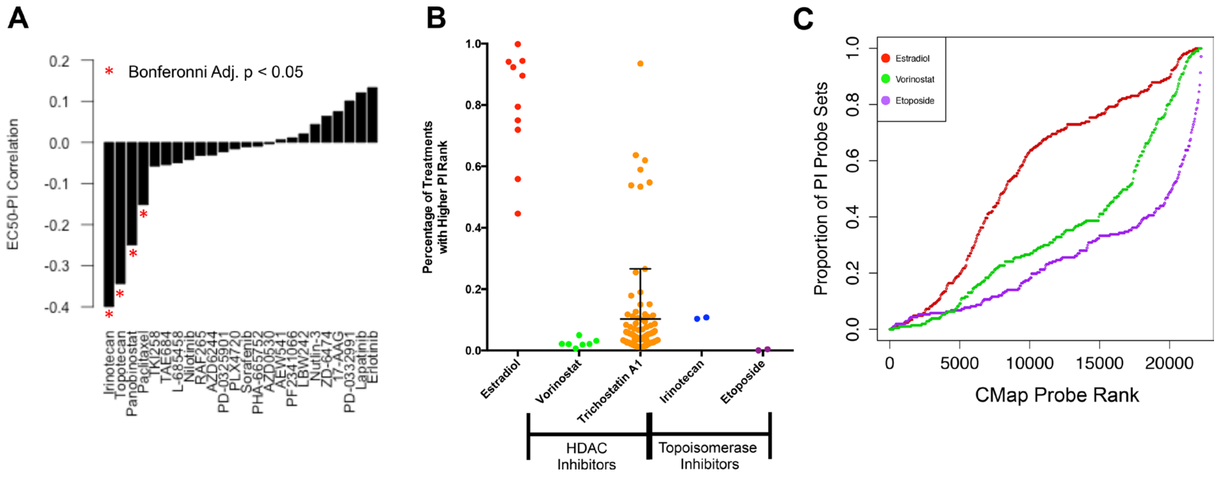


**Supplementary Figure 8:** (**A**) Spearman correlation of EC50 values and PI for 24 compounds across 486 cancer cell lines in the Cancer Cell Line Encyclopedia. Red stars indicate compounds whose correlation is significant ($p$-value < 0.05) after Bonferonni correction. (**B**) Treatment induced changes in proliferative index for compounds of interest. CMap rank corresponds to the relative magnitude of differential expression of a probe set after treatment with a compound of interest compared to a vehicle control with high rank corresponding to up-regulated genes and low ranks corresponding to down-regulated genes. PI rank was defined as the median rank of probe sets corresponding to PI genes. PI ranks are plotted as percentage of drugs ($n$ = 1309) possessing a higher PI rank or decrease in PI after treatment. Mean and standard deviation bars are plotted for Trichostatin A1 (**C**) Cumulative distribution plots for CMap rankings of probe sets corresponding to PI genes.

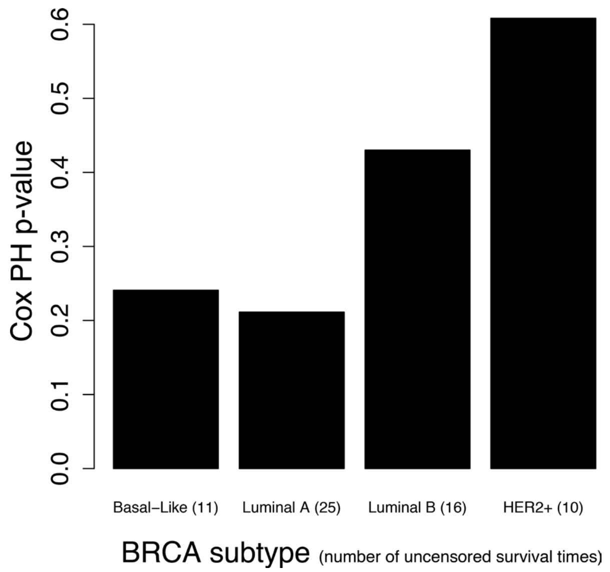


**Supplementary Figure 9: Tumor proliferative index Cox regression negative log *p*-values plotted by BRCA subtype with the number of censored survival times shown in parentheses.**

**Supplementary Table 1: Genes used to compute the proliferation index.** See Supplementary_Table_1

**Supplementary Table 2: TCGA cancers selected for study inclusion**

| Cancer_ID | Included in Analysis? | Total Patients | Uncensored Patients |
|---|---|---|---|
| ACC | Yes | 79 | 25 |
| BLCA | Yes | 385 | 101 |
| BRCA | Yes | 1038 | 102 |
| CESC | Yes | 393 | 56 |
| ESCA | Yes | 163 | 47 |
| GBM | Yes | 144 | 92 |
| HNSC | Yes | 508 | 164 |
| KIRC | Yes | 525 | 160 |
| KIRP | Yes | 266 | 29 |
| LAML | Yes | 148 | 96 |
| LGG | Yes | 463 | 74 |
| LIHC | Yes | 355 | 87 |
| LUAD | Yes | 493 | 123 |
| LUSC | Yes | 479 | 152 |
| MESO | Yes | 72 | 55 |
| OV | Yes | 252 | 144 |
| PAAD | Yes | 167 | 57 |
| SARC | Yes | 248 | 74 |
| STAD | Yes | 403 | 77 |
| **Total** | **-** | **6581** | **1715** |
| CHOL | No | 36 | 16 |
| COAD | No | 19 | 3 |
| DLBC | No | 46 | 5 |
| KICH | No | 66 | 9 |
| PCPG | No | 176 | 6 |
| PRAD | No | 482 | 7 |
| READ | No | 91 | 7 |
| SKCM | No | 87 | 9 |
| TGCT | No | 132 | 3 |
| THYM | No | 498 | 14 |
| UCEC | No | 164 | 11 |
| UVM | No | 77 | 13 |
| **Total** | **-** | **8455** | **1818** |

**Supplementary Table 3: GTEx tissues selected for study inclusion**

| SMTSD | Number of Samples |
|---|---|
| Adipose - Subcutaneous | 350 |
| Adipose - Visceral (Omentum) | 227 |
| Adrenal Gland | 145 |
| Artery - Aorta | 224 |
| Artery - Coronary | 133 |
| Artery - Tibial | 332 |
| Bladder | 11 |
| Brain - Amygdala | 72 |
| Brain - Anterior cingulate cortex (BA24) | 84 |
| Brain - Caudate (basal ganglia) | 117 |
| Brain - Cerebellar Hemisphere | 105 |
| Brain - Cerebellum | 125 |
| Brain - Cortex | 114 |
| Brain - Frontal Cortex (BA9) | 108 |
| Brain - Hippocampus | 94 |
| Brain - Hypothalamus | 96 |
| Brain - Nucleus accumbens (basal ganglia) | 113 |
| Brain - Putamen (basal ganglia) | 97 |
| Brain - Spinal cord (cervical c-1) | 71 |
| Brain - Substantia nigra | 63 |
| Breast - Mammary Tissue | 214 |
| Cells - EBV-transformed lymphocytes | 118 |
| Cells - Transformed fibroblasts | 284 |
| Cervix - Ectocervix | 6 |
| Cervix - Endocervix | 5 |
| Colon - Sigmoid | 149 |
| Colon - Transverse | 196 |
| Esophagus - Gastroesophageal Junction | 153 |
| Esophagus - Mucosa | 286 |
| Esophagus - Muscularis | 247 |
| Fallopian Tube | 6 |
| Heart - Atrial Appendage | 194 |
| Heart - Left Ventricle | 218 |
| Kidney - Cortex | 32 |
| Liver | 119 |
| Lung | 320 |
| Minor Salivary Gland | 57 |
| Muscle - Skeletal | 430 |
| Nerve - Tibial | 304 |
| Ovary | 97 |
| Pancreas | 171 |
| Pituitary | 103 |
| Prostate | 106 |
| Skin - Not Sun Exposed (Suprapubic) | 250 |
| Skin - Sun Exposed (Lower leg) | 356 |
| Small Intestine - Terminal Ileum | 88 |
| Spleen | 104 |
| Stomach | 192 |
| Testis | 172 |
| Thyroid | 323 |
| Uterus | 83 |
| Vagina | 96 |
| Whole Blood | 393 |

**Supplementary Table 4: Negative log10 Cox proportional hazards *p*-value of each gene for each cancer.** See Supplementry_Table_4

**Supplementary Table 5: GOrilla gene ontology terms enriched for survival associated transcripts in each cancer.** See Supplementry_Table_5

**Supplementary Table 6: GOrilla pathway enrichment for cross cancer survival associated genes.** See Supplementry_Table_6

**Supplementary Table 7: Samples used to train/test initial cross cancer prognostic models.** See Supplementry_Table_7

**Supplementary Table 8: Samples used to train/test initial PIC specific prognostic models.** See Supplementry_Table_8

**Supplementary Table 9: LASSO model coefficients for PIC cross-cancer model**

| Names | Coef | Description |
|---|---|---|
| (Intercept) | −0.104486543 | - |
| ADAM12 | 0.08585596 | Matrix Metalloproteinase |
| CKS2 | 0.037430983 | Regulatory cofactor to cyclin dependant kinase |
| CRYL1 | −0.087782597 | Member of cyrstallin family |
| DNA2 | 0.056431628 | DNA helicase |
| DONSON | 0.012986672 | Downstream neighbor of SON |
| HJURP | 0.098112224 | Histone chaperone |
| NDRG2 | −0.021927973 | Alpha/beta hydrolase |
| RAD54B | 0.031013034 | Helicase |
| SHOX2 | 0.039828771 | Homeobox protein |
| SUOX | −0.154971978 | Sulfite oxidation catalyst |

**Supplementary Table 10: PI correlation with somatic mutation burden**

| Cancer | Rho | *P*-value |
|---|---|---|
| GBM | 0.045 | 0.60 |
| OV | 0.092 | 0.25 |
| LUAD | 0.340 | 9.28E-06 |
| LUSC | 0.289 | 9.83E-05 |
| BRCA | 0.448 | 1.57E-37 |
| KIRC | 0.073 | 0.14 |
| LAML | 0.136 | 0.09 |
| HNSC | 0.129 | 0.02 |
| BLCA | 0.219 | 0.03 |
| All Cancers | 0.204 | 1.76E-23 |

**Supplementary Table 11: *P*-value for associations between gene mutation status and PI.** See Supplementry_Table_11
